# Penetrating corneal wound with traumatic cataract and intraocular foreign body-case report

**DOI:** 10.22336/rjo.2017.10

**Published:** 2017

**Authors:** Dorin Căciulă, Monica Gavriș, Irina Tămășoi

**Affiliations:** * ”Dr. Constantin Papilian” Military Emergency Hospital, Cluj-Napoca, Cluj, Romania; ** Dej City Hospital, Dej, Romania

**Keywords:** corneal wound, intraocular foreign body, cataract, iris-claw intraocular lens

## Abstract

Open globe injuries complicated with the presence of an intraocular foreign body mostly affect young males and represent a vision threatening condition.

We presented the case of a 48-year-old male who presented to our emergency service due to ocular pain and blurred vision in his right eye.

A metallic foreign body situated between 1 and 12 o’clock, near the corneoscleral limbus, that perforated the cornea, the iris, the anterior capsule of the lens and the lens, was detected at the slit-lamp examination. We decided to immediately remove the foreign body that was approximately 20 mm long. The following day, traumatic cataract had already developed, so we performed cataract extraction. Despite the dimensions of the intraocular foreign body, the retina was attached and there were no sign of retinal tears or vitreous haemorrhage.

The proper management in this case led to good results in spite of the dimensions of the intraocular foreign body.

**Abbreviations:** IOFB = Intraocular Foreign Body, IOL = Intraocular Lens, PVR = Proliferative Vitreo-Retinopathy

## Introduction

The incidence of ocular trauma is relatively common despite the anatomical and functional protective mechanisms of the eye. The orbital rim prevents many direct injuries from affecting the eye, and reflex closure of the lids aids in insulating the globe [**1**].

Open globe injuries complicated with the presence of an intraocular foreign body represent a vision threatening condition [**2**]. 

Patients may have an intraocular foreign body without being aware that the eye was penetrated. A history of hammering metal on metal should alert the clinician to search for an intraocular foreign body. The material will dictate the type of reaction: silver, aluminum, platinum, and gold are rather inert and cause little reaction, but iron can lead to siderosis bulbi and eventual loss of the eye [**1**].

Surgical intervention with or without pars plana vitrectomy combined with intraocular foreign body removal and cataract extraction may preserve severely traumatized eyes and maintain or even improve vision [**2**].

The choice of the type of cataract surgery performed in such cases depends on the surgeons’ experience and the particularity of the case. Frequently traumatic cataract is associated with anterior or posterior luxation or subluxation of the lens, anterior or posterior capsular rupture, ectropion uveae, corneal wounds, vitreous in the anterior chamber, vitreous, or iris loss. When facing a very complex case it is advisable to restore the anatomical integrity of the eye first and perform cataract surgery later when we have the comfort of a stable anterior chamber.

Visual recovery after ocular trauma also depends on the involvement of the retina. The extraction of the opacified lens has a great importance as it allows the vitreoretinal surgeon to detect and cure associated retinal complications.

Besides the right management of the corneal or scleral wound, retinal complications and traumatic cataract, another important step in managing these patients is the choice of the type of artificial intraocular lens.

There are several options in placing the intraocular lens: in the posterior chamber - iris fixation, scleral suture, placing it in the sulcus or in the anterior chamber. It is advisable to place the artificial IOL in the posterior chamber.

Transscleral fixation of a posterior chamber IOL is technically challenging, requiring more surgical time and has an increased possibility of associated complications such as retinal detachment, IOL decentration and endophthalmitis. Angle – supported anterior chamber IOLs are associated with long-term complications such as bullous keratopathy.

Posterior iris-claw fixated IOL is a viable option due to less surgical time and minimal complications. Progressive pigment dispersion and secondary pigmentary glaucoma are not a common late complication of this type of IOL [**3**-**5**].

## Case report

Herein we presented the case of a 48-year-old male who presented to our emergency service due to ocular pain and blurred vision in his right eye. While cleaning an oven with a metallic brush, the patient felt a foreign body sensation in the right eye. Best-corrected visual acuity of his right eye was 0.9.

**Fig. 1:**
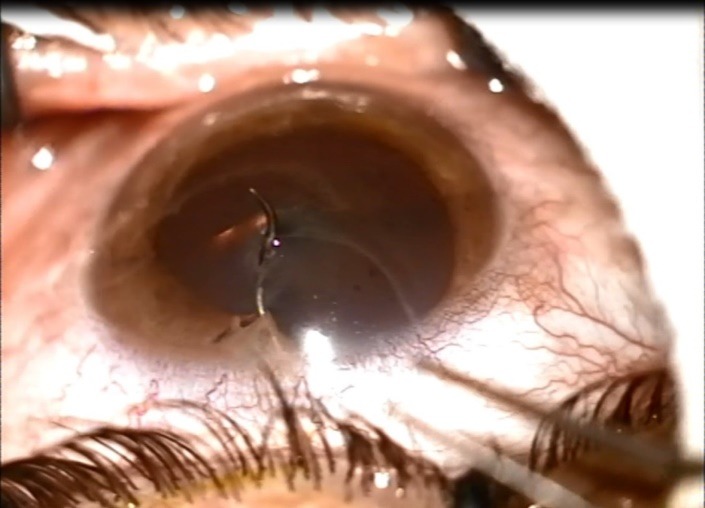
IOFB at presentation

Slit lamp examination of the anterior pole of the right eye revealed conjunctival congestion, watery discharge. Around 12 o’clock, a metallic foreign body was detected near the corneoscleral limbus, which perforated the cornea, the iris, the anterior capsule of the lens and the lens (**Fig. 1**). We could not perform the full examination of the posterior pole, so we could not establish the trajectory of the intraocular foreign body. Ocular B-scan was not performed because of the penetrating corneal wound. X-ray of the orbit did not offer enough details and computed tomography could not be performed at that time in our service.

**Fig. 2 a,b:**
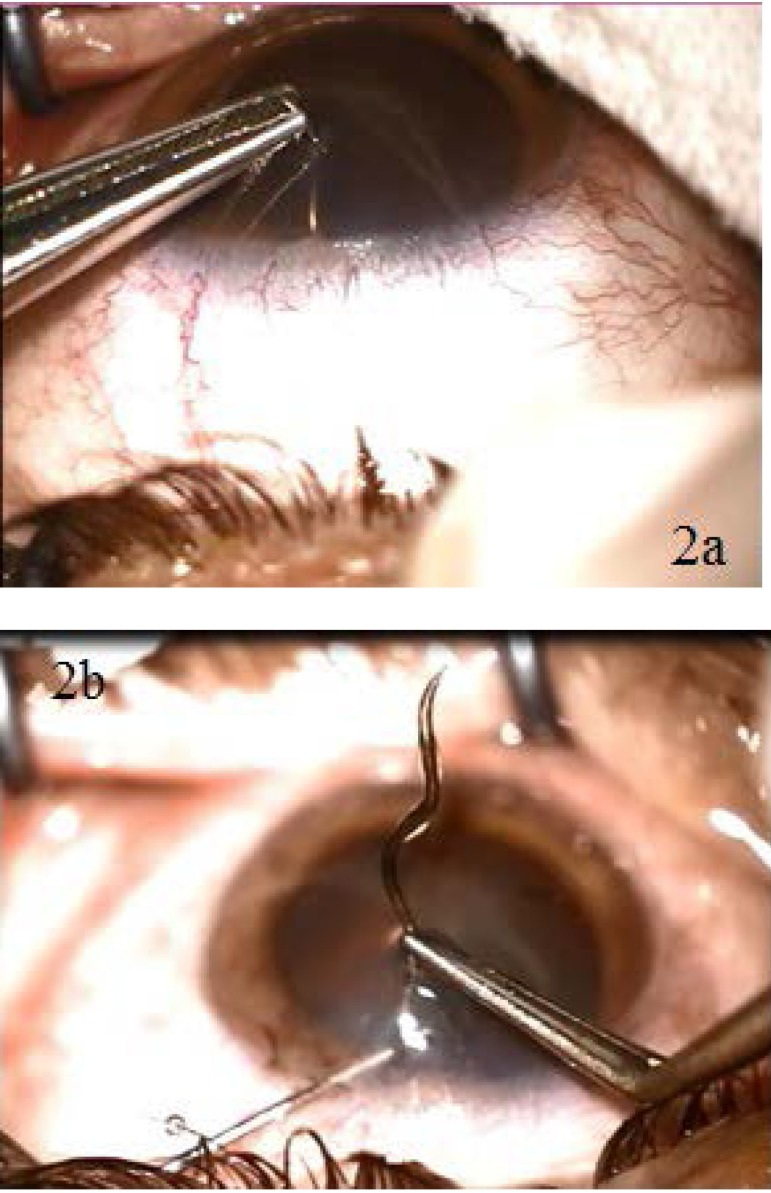
Intraoperative aspects - extraction of the helicoidal IOFB with the forceps

**Fig. 3:**
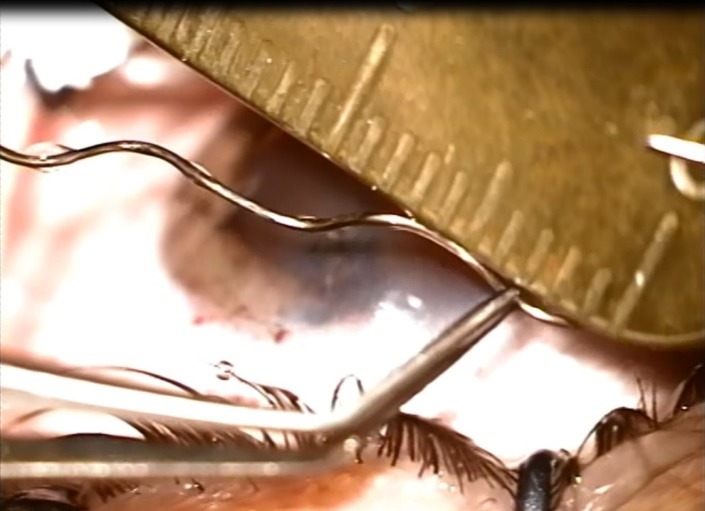
IOFB measuring almost 20 mm in length

After performing an anti-tetanic prophylaxis, we decided to extract the intraocular foreign body by using a forceps. Since it had a helicoidal shape, we had to perform several circular movements (**Fig. 2 a,b**). We were surprised to find out that the wire that perforated the eye was almost 20 mm long (**Fig. 3**). We injected an antibiotic in the anterior chamber and placed a contact lens to protect the cornea and facilitate the healing of the point-like corneal wound

**Fig. 4:**
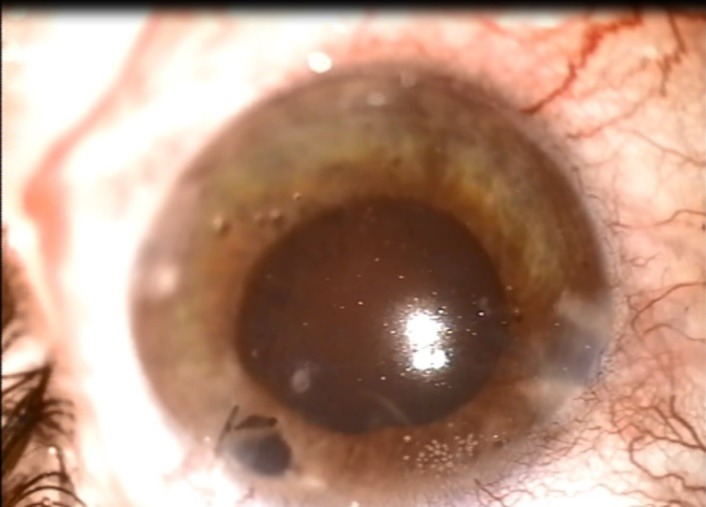
Postoperative aspect (after cataract extraction). We noticed the round pupil and the iris defect between 12 and 1 o’clock

On the first day postoperatively, the slit lamp examination revealed a stable anterior chamber and a totally opacified lens with anterior capsule rupture. B-scan ultrasound showed no sign of vitreous haemorrhage and an attached retina. Visual acuity of the right eye was hand motion determined because of the traumatic cataract. We decided to extract the opacified lens. Due to the age of the patient, the lens was very soft so we performed its extraction with a blunt cannula under viscoelastic protection, with good results (**Fig. 4**). We noticed a posterior capsule break because the foreign body passed through the lens into the vitreous cavity. Surgical aphakia was corrected with an intraocular lens fixated to the posterior face of the iris. At one week follow-up, the best corrected visual acuity was 0.8 and the retina was attached.

## Discussion

Penetrating corneal wounds with intraocular foreign bodies are challenging situations, as they require very complex therapeutic management. Sometimes, the surgeon can extract the foreign body and cure the associated complications in one surgical intervention, but there are times when several interventions are needed. In our case, we performed the extraction of the intraocular foreign body and the management of the corneal wound at once, postponing the cataract extraction and IOL implantation.

A thorough examination of the patient with penetrating corneal wound should be made as most of the times one or several intraocular foreign bodies may be associated. Patel et al. suggested that 14% of the patients with penetrating globe trauma have intraocular foreign bodies. Radiological examinations can be performed to detect intraocular foreign bodies: X-ray, ultrasound, computed tomography of the orbit. CT examination at presentation identified IOFB in more than 90% of the cases, B-scan ultrasound revealed an IOFB in 51.9% of the cases and clinical eye examination in 45.6% of the cases [**6**,**7**]. In our case report, the intraocular foreign body was visible at the clinical examination. CT or B-scan ultrasound could have revealed the depth and complications associated with the IOFB: vitreous hemorrhage, retinal detachment, and endophthalmitis.

A B-scan ultrasound was performed the day after the IOFB extraction. The retina was attached and the vitreous was clear and homogeneous.

Another important fact is the management of the cornel perforation. In our case, it was point-like, with the diameter of less than 1 mm. After extracting the intraocular foreign body, the anterior chamber was stable. That was why we decided to place a contact lens instead of a corneal suture, avoiding the irregular astigmatism and the corneal scar due to the suture. 

Most of the patients with intraocular foreign bodies already have traumatic cataract when they address to the ophthalmology service. In our case, since we saw our patient one hour after the accident, the lens was clear. Traumatic cataract developed later, after IOFB extraction.

We decided to extract the opacified lens. This led to the improvement of the visual acuity and to a better visualization of the posterior pole, which helped in the detection and treatment of retinal complications [**8**].

There are several methods of treating traumatic cataract: extracapsular, intracapsular extraction and phacoemulsification. Because of the anterior capsule tear, in our case, capsulorhexis was more challenging than usual. We also had to pay attention to hydrodissection because posterior capsule tear might also be present. Ocular hypotony and posterior capsule break makes the surgical intervention difficult and risky [**9**].

**Fig. 5 a,b,c:**
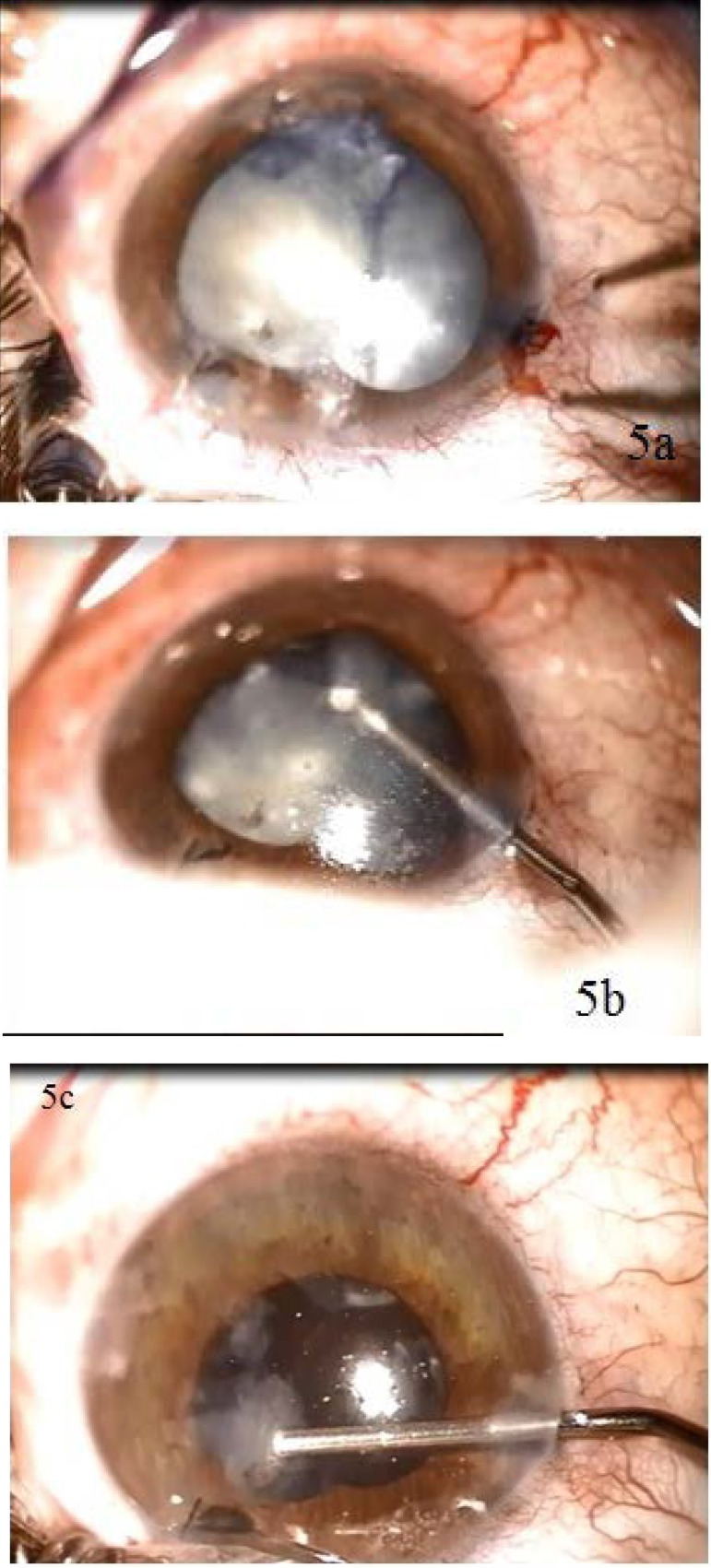
Intraoperative aspects: extraction of the traumatic cataract

In this particular case, the traumatic cataract was intumescent. This led to the extension of the anterior capsule break caused by the IOFB. We used trypan blue to stain the anterior capsule. Capsulorhexis was followed by gentle hydrodissection and extraction of the soft lens with a cannula (**Fig. 5 a,b,c**). Repeated viscoelastic injections were performed in the anterior chamber during the procedure in order to maintain its stability and protect the corneal endothelium. Vitreous could be found in the anterior chamber due to a posterior capsule break. Triamcinolone straining and anterior vitrectomy were used for its removal.

Choosing the right type of intraocular artificial lens is very important when appropriate capsular support is absent. Several types are available: IOL with scleral fixation, anterior chamber IOL and iris-claw IOL. Several studies revealed that fixating an iris-claw IOL on the posterior face of the iris result in reduced surgical time and good stability of the IOL on long-term.

According to Labeille et al., the complications associated with this type of IOL were: cystoid macular edema, retinal detachment, transient intravitreal hemorrhage, secondary glaucoma and choroidal detachment, but with a lower incidence in comparison with the scleral fixation IOL [**10**]. Placing the IOL on the posterior face of the iris reduces the risk of endothelial cells loss and of bullous keratopathy. These are some of the reasons that determined us to use this type of IOL to correct the surgical aphakia in our case. Postoperative best-corrected visual acuity was 0.8.

Even if surgical outcome was good, long-term complications could arise.

The most important complication of open globe injury is endophthalmitis. The frequency of endophthalmitis after open globe injury is 6.8% [**11**] and the etiology is mostly Staphylococcus spp. The following factors were associated with the subsequent development of endophthalmitis: dirty wound, retained intraocular foreign body, lens capsule breach, delayed primary repair [**11**]. Our patient underwent a prompt surgical intervention for IOFB extraction followed by the irrigation of the anterior chamber with vancomycin and systemic antibiotic therapy. It did not develop any signs or symptoms of endophthalmitis.

Secondary glaucoma is another complication. There is a strong correlation between traumatic cataract, angle recession of minimum 180 degrees, iris trauma, traumatic ectopia of the lens and secondary traumatic glaucoma [**12**]. According to Bojikian et al., traumatic IOP elevation and glaucoma are common after visually salvageable open-globe injury. Most cases develop within 6 months, although longer follow-up remains important for case detection [**13**].

Between 15-32% of the patients with IOFB may develop retinal detachment [**8**]. In our case report, retinal injury was not associated even though the IOFB was almost 20 mm long.

Young patients can develop proliferative vitreoretinopathy in the absence of retinal breaks, leading to vitreoretinal tractions, secondary retinal breaks, tractional retinal detachment, and decreased visual acuity. It is important to perform a thorough follow up of young patients with open globe injury and address them to the vitreoretinal surgeon at the first signs of PVR.

Macular pucker is frequent when the IOFB touches the retina near the macula or the temporal vascular arcades [**14**]. Vitreous hemorrhage is present in almost all the cases of IOFB following open globe injury. Sometimes, it reabsorbs by itself but most of the times it requires posterior vitrectomy.

We must not neglect the risk of sympathetic ophthalmia. In our case, the risk was low because of the good surgical management without vitreous or iris loss.

## Conclusions

Ocular trauma occurs mostly in young, active males.

Open globe injuries with IOFB require a thorough examination of the eye. The detection and treatment of the associated complications can determine a favorable outcome with the restoration of the anatomical integrity of the eye and a good visual acuity.
